# Overexpression of the *Capebp2* Gene Encoding the PEBP-like Protein Promotes the Cap Redifferentiation in *Cyclocybe aegerita*

**DOI:** 10.3390/jof9060657

**Published:** 2023-06-12

**Authors:** Bopu Cheng, Nan Tao, Yuanhao Ma, Hongmei Chai, Ping Liu, Weimin Chen, Yongchang Zhao

**Affiliations:** 1 College of Life Science, Southwest Forestry University, Kunming 650224, China; cpb1997@sina.com; 2 Biotechnology and Germplasm Resources Institute, Yunnan Academy of Agricultural Sciences, Kunming 650223, China; tn1953@126.com (N.T.); jianxyjian@126.com (Y.M.); chm621@aliyun.com (H.C.); liuping0606@126.com (P.L.); 3 Yunnan Provincial Key Laboratory of Agricultural Biotechnology, Kunming 650223, China; 4 Key Laboratory of Southwestern Crop Gene Resources and Germplasm Innovation, Ministry of Agriculture, Kunming 650223, China

**Keywords:** PEBP, cap redifferentiation, *Cyclocybe aegerita*, overexpression, fruiting body development

## Abstract

Phosphatidylethanolamine-binding protein (PEBP) is widely involved in various physiological behaviors, such as the transition from vegetative growth to reproductive growth in plants, tumorigenesis in the human, etc. However, few functional studies have examined *pebp* genes affecting the development of fungi. In this study, *Capebp2* was cloned from *Cyclocybe aegerita* AC0007 strains based on the genome sequence and gene prediction, and the sequence alignment of CaPEBP2 with other PEBP proteins from other biological sources including plant, animal, fungi, and bacteria indicated that PEBP had low sequence similarity in fungi, whereas all protein sequences had some conserved motifs such as DPDAP and HRY. Expression analysis showed the transcription level of *Capebp2* increased approximately 20-fold in fruiting bodies compared with mycelia. To uncover the function of *Capebp2* in *C. aegetita* development, *Capebp2* was cloned into a pATH vector driven by the *actin* promoter for obtaining overexpression transformant lines. Fruiting experiments showed the transformed strains overexpressing *Capebp2* exhibited redifferentiation of the cap on their surface, including intact fruiting bodies or partial lamella during fruiting development stage, and the longitudinal section indicated that all regenerated bodies or lamella sprouted from the flesh and shared the epidermis with the mother fruiting bodies. In summary, the sequence characterization of *Capebp2*, expression level during different development stages, and function on fruiting body development were documented in this study, and these findings provided a reference to study the role of *pebp* in the development process of basidiomycetes. Importantly, gene mining of *pebp*, function characterization, and the regulating pathways involved need to be uncovered in further studies.

## 1. Introduction

Phosphatidylethanolamine-binding protein (PEBP) is a class of regulatory proteins that widely exist in bacteria, fungi, plants, and animals. It was originally isolated from the bovine brain [1] and named PEBP1 due to its preferred binding to phosphatidylethanolamine [2]. In mammals, the PEBP2 and PEBP4 proteins have been successively discovered based on their functional characteristics, although the transcript of *pebp3* is not found in the tested species and is presumed to be a pseudogene [3].

PEBP1 is widely involved in various cellular physiological behaviors, including cell growth and differentiation [4], cell cycle [5], and genome stability [6]. In plants, the functions and molecular regulatory pathways of the PEBP protein family have been thoroughly elucidated, with the most important function of this protein family being participation in plant flowering. A previous study reported PEBP as a florigen affected by the light cycle, which could be transferred from leaves to the stem tip meristem, thus promoting flower formation [7]. Subsequent studies on multiple species of the florigen indicated it was a homologous and conserved flowering signaling molecule [8] and named it the PEBP family flowering locus (FT).

PEBP family proteins also exhibit various physiological regulatory effects in plants, which are mainly related to flower formation and can be divided into three groups based on their function: the flowering locus T (FT) group, the terminal flower 1 (TFL1) group, and the mother of FT and TFL1 (MFT) group [9,10]. The protein encoded by FT is a plant floral hormone that promotes floral development [11,12,13], whereas TFL1 has an opposite function, and both FT and TFL1 control flower formation in the forward and reverse directions [14]. MFT, an ancestor of the evolutionary relationship between FT and TFL, also plays an important role in seed germination and development [15]. The three above-mentioned group proteins have been found in various spermatophytes, whereas in some cryptogams, such as moss, only a portion of MFT is present, and FT and TFL proteins are lacking [16]. In the model plant *Arabidopsis thaliana*, there are six PEBP proteins, among which the FT group includes two proteins, namely, FT and TSF (twin sister of FT), both of which can promote flower formation. However, mutations in genes encoding these two proteins can cause delayed flowering [17], with both showing similar molecular regulation mechanisms [18]. The FT-like protein, activated by the zinc finger protein CONSTANS (CO), is transported to the top meristem of branches and interacts with the transcription factor FD to activate downstream target genes such as AP1 of floral division tissue, thus promoting floral development [12,19]. The TFL1 group includes TFL1, FT-like protein BFT (brother of FT), and CEN (centroradialis) proteins [14]. MFT only has MFT-like proteins that are involved in the ABA signaling pathway of abscisic acid [20]. Moreover, systematic studies on the function of the PEBP protein in multiple species, including maize [9], pea [21], rice [13], barley [22], *Populus* [23], tomato [24], sugar beet [25], soybean [26], and potato [27], were conducted to confirm the roles of PEBP protein family members in the transition from vegetative growth to reproductive growth in plants.

In addition to participating in the regulation pathway of plant flower formation, PEBP family proteins are also involved in the regulation of plant hormones, including strigolactone, cytokinin, auxin, brassinosteroid (BR), jasmonic acid, and abscisic acid (ABA) pathways [28,29,30,31]. In *Arabidopsis*, direct and indirect targets of TFL1–FD suggest that TFL1–FD blocks auxin signaling and response according to the targeted gene characteristics [31]. In tomatoes, loss-of-function mutants of the ortholog of TFL1, SELF PRUNING (SP), exhibit traits such as shoot determinacy, early flowering, and simultaneous fruit ripening [32]. Further analysis shows polar auxin transport and responses are altered in *sp* mutants [33], and DNA affinity purification sequencing and expression analysis in mutants shows FD homologs linking to the OsARF19 auxin-responsive transcription factor, supporting the link from PEBPs to auxin response [34]. In *Setaria*, TFL1–FD-repressed genes are involved in the BR signaling [31], and BR biosynthesis mutants cause formation of additional spikelets, suggesting that BR blocks inflorescence branching [35]. In addition, the accumulation of the BR early response regulator and basic helix-loop-helix (bHLH) transcription factor promotes flowering by up-regulating FT expression under blue light irradiation [36]. Multiple genes in the abscisic acid pathway are also common targets for TFL1, FD, and the FD homologous FDP [30,31], and abscisic acid up-regulates FT expression and accumulation [37,38]. The loss of MFT and FD or FDP function cause the ABA-dependent phenotypes during cotyledon greening and seed germination stages [20,39]. These studies directly or indirectly indicate that PEBP family genes are widely involved in the main regulatory pathways in plant physiological development, targeting important growth nodes such as seed germination, apical tissue differentiation, and sexual reproduction.

Genomics has generated considerable data, including genome maps of multiple species of fungi, which annotate several PEBP proteins. However, the function of PEBP in fungi remains unknown. As an important commercial mushroom, *C*. *aegerita* is a delicious species with high nutrient value. It is a typical basidiomycete species with a short life cycle, which renders this species important in fruiting body development research. In this study, a gene encoding PEBP named *Capebp2* of *C. aegerita* 0007 was cloned based on genome annotation, and the sequence characterization was performed compared with the homology genes in other species. Functional analysis of *pepb2* was conducted by the overexpression of *Capebp2* in the *C. aegerita* 0007 strain, and the morphologies of transformants were compared with those of wild strains during various developmental stages, providing new insights to understand the functions of PEBP in fungi.

## 2. Materials and Methods

### 2.1. Strains and Culture Conditions

*C. aegerita* dikaryotic strain AC0007 was employed for the experiment in this study. The mycelia were cultivated on a YPD medium plate (0.2% yeast extract, 0.2% peptone, 2% dextrose, and 1.5% agar) at 25 °C. Three agar disks (1 cm diameter each) were inoculated in a sawdust medium (64% hardwood sawdust, 15% wheat bran, 1% plaster, and 20% cottonseed shells) to produce fruiting bodies. Two batches of fruiting tests for transformed strains were separately conducted in a greenhouse or with a thermostat with constant humidity. *E. coli* DH5α strain used for plasmid amplification was cultured in Luria–Bertani (LB) medium containing 100 µg/mL ampicillin.

### 2.2. Capebp2 Cloning and Sequence Analysis

The genomic DNA of AC0007 was extracted from mycelia using the fungal gDNA isolation kit (Biomiga, San Diego, CA, USA). Total RNA was prepared from the tissues using the RNAiso reagent (TaKaRa, Dalian, China), and single-stranded cDNA was synthesized from 0.75 µg of total RNA using the PrimeScript TM RT reagent kit (TaKaRa, Kyoto, Japan). The complete DNA and cDNA products of *Capebp2* were obtained using the primer pairs capebp2F/R (Table 1) based on the genome information of AC0007. These products were then cloned into the PMD19T vector for sequencing. For phylogenetic analyses, multiple protein sequence alignments were performed by the program MUSCLE in the software MEGA6 v6.06 [40] using default parameters, and the phylogenetic trees for the PEBP sequences were inferred using the neighbor-joining (NJ) method. PEBP sequences from species of plants, animals, fungi, and bacteria were obtained by blasting CaPEBP sequences against the NCBI GenBank database.

### 2.3. Vector Construction and Transformation

The overexpression vector was constructed and transformed using the method described by a previous study [41]. In brief, The pATH vector contains the CaMV35S terminator, the hygromycin B phosphotransferase gene (*HygR*), the *gpd* promoter from *Ustilago maydis* (*Pum-gpd*), and *C. aegerita actin* promoter (*Ca-actin*) for driving the overexpression of *HygR* and the targeted gene, respectively. The amplified products of *Capebp2* cDNA using the primer pairs capebp2F/R (Table 1) were ligated into the *EcoRV* enzyme-digested site between the *pCa-actin* and T35s elements of the pATH vector. The constructed vector pATH–pebp2 was transferred to DH5α for plasmid amplification. The genetic transformation was conducted using the PEG-mediated method, as described previously [28]. The mycelia were digested using a lysing enzyme solution (1% cellulase R-10 (Yakult Pharmaceutical Industry Co., Ltd, Tokyo, Japan), 1% Lywallzyme (Biktak Bio Tech, Beijing, China), and 1% lysing enzyme (Sigma-Aldrich, St. Louis, MI, USA) in a 0.6 M mannitol buffer), following which the protoplasts were washed in STC solution (1.2 M sorbitol, 10 mM CaCl_2_, and 50 mM Tris-HCl, pH 7.0) to mix them with plasmid DNA for transformation. The reaction mixture was added to a YPD regeneration medium (20.5% sucrose, 0.2% tryptone, 0.2% yeast extract, 1% agar), following which a YPD medium containing 150 µg/mL hygromycin B was poured over the plates to screen transformants after five days. 

### 2.4. Transformant Verification and Expression Analysis

The regenerated strains were transferred to a YPD medium containing 120 µg/mL hygromycin B to further verify strain resistance. Primer pairs 19 ha3/19 ha4 anchored to the flanking region of the inserted fragment were used for PCR validation (Table 1). The positive transformants were deposited in a YPD medium containing 50 µg/mL hygromycin B as the selection pressure. All strains and plasmids were deposited in our laboratory.

The qPCR reaction was performed in a CFX96 real-time PCR detection system (Bio-Rad). The *gpd* gene was used as the internal reference gene for qPCR. The primer pairs gpd qF/R and pebp qF/R were used for the amplification of *gpd* and *Capebp2*, respectively (Table 1). The reaction system included 10 µL of iTaqTM universal SYBR green SuperMix (Bio-Rad Laboratories, Hercules, CA, USA), 1 µL of upstream primer, 1 µL of downstream primer, and 8 µL of ultrapure water, in a total volume of 20 µL. Each sample was analyzed in triplicate. The reaction conditions were as follows: initial polymerase activation at 95 °C for 20 s, followed by 40 cycles of 95 °C for 5 s and 60 °C for 30 s. Each sample was performed in triplicate. Glyceraldehyde-3-phosphate dehydrogenase (GPD) was used as the reference gene for the normalization of the qPCR data. The gene expression levels were calculated using the 2^−ΔΔCT^ method.

### 2.5. Data Analysis

The compilation and mapping of experimental data were performed using Graphpad prism5 software. One-way analysis of variance (ANOVA) and the least significant difference (LSD) test were used to examine the significant differences among samples. All experiments were conducted in triplicate for data precision.

## 3. Results

### 3.1. Sequence Analysis of Capebp2

**Figure 1:**
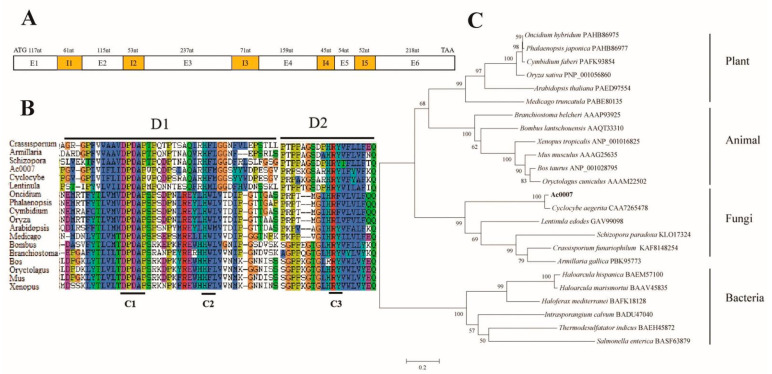
Sequence analysis of *Capepb2*. (**A**) Sequence structure of *Capebp2*. E1-E6 represent exons, and I1-I5 represent introns. (**B**) The conserved protein sequence of CaPEBP2 (Ac0007) and its comparison with sequences from other strains. D1-2 represent the conserved regions. C1-3 was highly conserved motifs. (**C**) The phylogenetic tree for CaPEBP2 with other PEBP proteins from plants, animals, fungi, and bacteria. PEBP sequences from species of plants, animals, fungi, and bacteria were obtained by blasting CaPEBP sequences against the NCBI GenBank database.

Based on the genome sequence and gene prediction, the primer pairs capebp2F/R were used to amplify the complete DNA and cDNA sequences. Sequence analysis revealed that *Capebp2* was 1182 bp in length and contained five introns and six exons encoding 299 aa (Figure 1A). The sequence alignment of CaPEBP2 with other PEBP proteins from other biological sources indicated that PEBP had low sequence similarity in fungi, whereas all protein sequences had three conserved motifs (C1–C3) in the D1 and D2 regions (Figure 1B). The phylogenetic analysis of the PEBP protein sequences indicated that species from plants, animals, fungi, and bacteria were divided into independent branches. Among them, proteins from plants and animals were clustered together and further clustered with those of fungi (Figure 1C). In the clade of fungi, the sequence of CaPEBP2 was closest to that of *C. aegerita* (CAA7265478), indicating that both shared high similarity (Figure 1C). The DNA and protein sequences of *Capebp2* are shown in Supplementary File S1.

### 3.2. Characterization of the Expression of Capebp2 during the Development of C. aegerita

**Figure 2:**
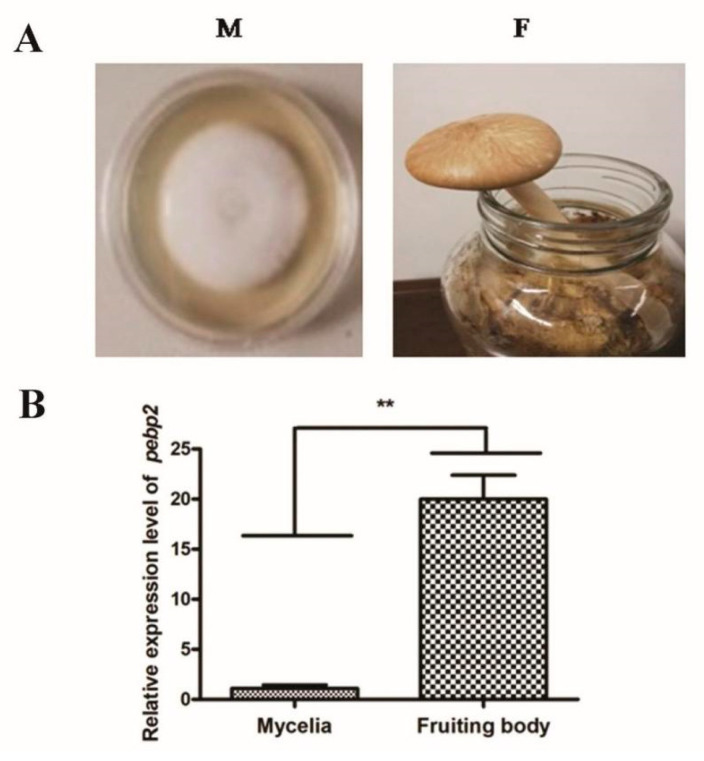
Expression characterization of *Capepb2* in mycelia and fruiting bodies. (**A**) The mycelia (M) and fruiting bodies (F) of the Ac0007 strain. (**B**) The expression level of *Capebp2* in mycelia and fruiting bodies. Asterisks (**) indicate data that differed significantly based on a *p*-value of <0.001 (*t*-test) as the significance threshold.

To characterize the expression of *Capebp2* in different developmental stages, mycelia and fruiting bodies were collected to prepare for RNA extraction. The primer pairs Pebp qF/R were used to determine the expression of *Capebp2*, and Gpd qF/R was used as the reference gene for the normalization of the qPCR data. The results revealed that the expression level increased approximately 20-fold in fruiting bodies (Figure 2).

### 3.3. Validation of Transformants and Expression of Capebp2

**Figure 3:**
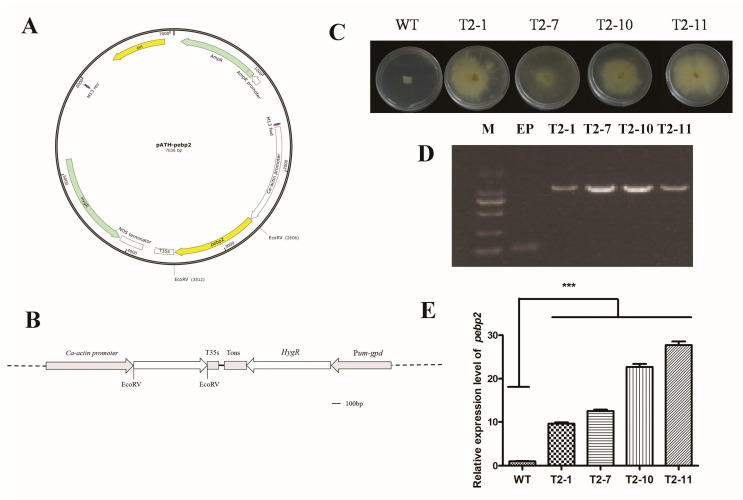
Plasmid construction, transformants verification, and expression analysis of *Capebp2.* (**A**) Structure of the pATH–pebp2 plasmid. *HygR* (hygromycin B phosphotransferase gene); *Ca-actin* promoter (*C. aegerita actin* promoter) (**B**) Diagram of the overexpression vector construction. The complete fragment of *Capebp2* was inserted at the site between the *Ca*-*actin* promoters and T35s. *HygR* (hygromycin B phosphotransferase gene); *Ca-actin* (*C. aegerita actin* promoter) (**C**) The resistance test of WT and transformants in the YPD medium containing 150 µg/mL hygromycin B. (**D**) The PCR verification of transformants. M, DL 2000 marker, EP, empty plasmid without target gene. (**E**) The expression analysis of *Capebp2* in the mycelia of WT and transformants. Asterisks (***) indicate data that differed significantly based on a *p*-value of < 0.0001 (*t*-test) as the significance threshold.

The overexpression transformants were obtained by constructing a pATH–pebp2 vector driven by the *Ca-actin* promoter (Figure 3A,B). The transformants were determined through hygromycin screening and PCR verification. The regeneration strains were transferred to a medium containing hygromycin B for analysis of resistance. Four strains, namely, T2–1, T2–7, T2–10, and T2–11, showed significant resistance to hygromycin B, whereas the growth of wild-type strains was completely inhibited (Figure 3C).

The resistance transformants were further verified by PCR amplification using the specific primer pair adhF and adhR targeting the *actin* promoter and T35s regions, and the sequencing results revealed that they were all positive transformants (Figure 3D). The mycelia of four transformants were collected for extraction of total RNA, and an equal amount of RNA was used for cDNA synthesis. The expression of *Capebp2* in all four transformants increased to a high level of approximately 9.60-fold, 12.53-fold, 22.69-fold, and 27.74-fold in T2–1, T2–7, T2–10, and T2–11, respectively, compared to that in the WT strain (Figure 3E).

### 3.4. The Effect of Overexpression of Capebp2 on the Fruiting Bodies

The mycelia of WT strains and the four transformants were inoculated in a sawdust medium at 25 °C for 40 days and then transferred to a greenhouse or thermostat with constant humidity for fruiting. The plasmid detection was conducted in fruiting bodies of four transformants using the specific primer pair adhF and adhR targeting the *actin* promoter and T35s regions for PCR verification, and the result showed the plasmids were all present in tested samples (Supplementary File S2).

**Figure 4:**
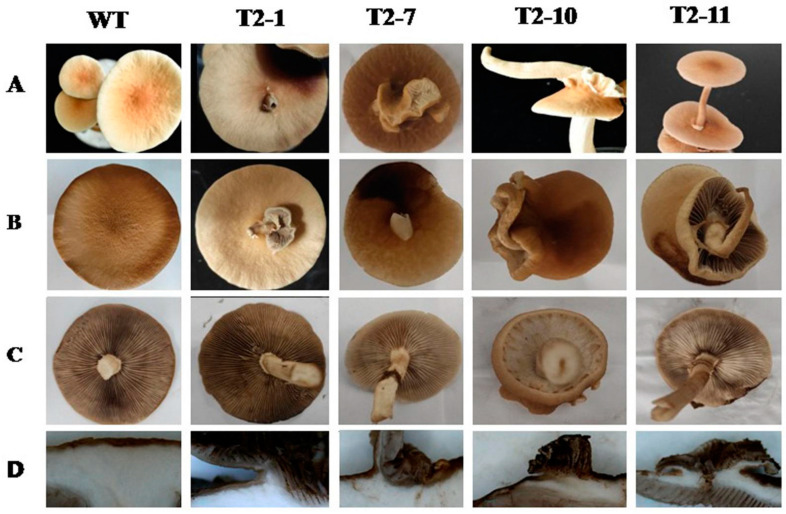
Morphology of the fruiting bodies in WT, T2–1, T2–7, T2–10, and 2-T11. (**A**,**B**) Cap redifferentiation of fruiting bodies. (**C**) The reverse side of fruiting bodies. (**D**) Longitudinal section image of fruiting bodies.

All fruiting bodies of *Capebp2*-overexpression strains exhibited redifferentiation of the cap on their surface (Figure 4A–C). These fruiting bodies were of two types: (1) complete fruiting bodies in T2–1, T2–10, and T2–11, among which the regeneration fruiting bodies sprouted from the flesh of fungi with a stalk or cap; and (2) partial lamella without stalk in T2–1 and T2–7, among which the cap surface sprouted partial lamella and the longitudinal section indicated that all regenerated bodies or lamella sprouted from the flesh and shared the epidermis with the mother fruiting bodies.

**Figure 5:**
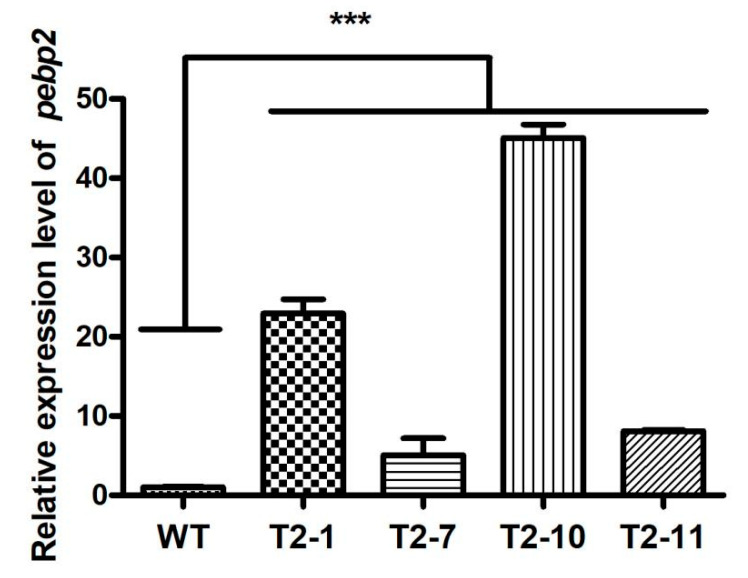
The expression level of *Capebp2* in mature fruiting bodies of WT and transformants T2–1, T2–7, T2–10, and 2-T11. Asterisks (***) indicate the data that differed significantly based on a *p*-value of <0.0001 (*t*-test) as the significance threshold.

To examine the expression level of *Capebp2* in fruiting bodies, the tissues in the regeneration site were sampled for cDNA synthesis. *Capebp2* in fruiting bodies also showed a high expression level in four transformants, approximately 22.94-fold, 5.07-fold, 45.08-fold, and 8.12-fold in T2–1, T2–7, T2–10, and T2–11, respectively, compared to the level in the WT strain (Figure 5).

## 4. Discussion

The function of the PEBP protein family has been thoroughly studied in plants and is known to finely regulate flower formation under the regulation of light signals [14,31,42]. It also plays a regulatory role in endosperm development [43], tuber and bulb formation, and bud development [44,45]. However, the function of this gene family has not been studied in fungi. *C*. *aegerita* is a delicious species widely cultivated in eastern Asia, with high nutrient value. Importantly, its short life cycle renders this species as an ideal species for studying the development of fruiting bodies. In the previous study, we screened some genes highly expressed at the fruiting body stage through transcriptome analysis, one of which was annotated as *pebp* gene. However, the protein sequence of it was quite different from that of PEPB gene in other species such as plants; we further analyzed the sequence characteristics and function of this gene in *C. aegerita*.

The PEBP family protein sequence is highly conserved in plants and animals [9,12,17,26], and is easily found through omics analysis. However, it has not been explored in fungi for a long time, mainly due to the significant differences in sequence characteristics between animals and plants, which affects the finding of PEBP sequences in fungi. In this study, systematic analysis revealed that the PEBP protein had high similarity in plants, animals, fungi, and bacteria, and was divided into four clades in the phylogenetic tree. Sequence alignment indicated that all PEBP protein sequences had several conserved motifs, including DPDAP and HRY, thus providing a reference for the identification of PEBP sequences in other species. In plants, PEBP gene numbers ranges from 6–8 to nearly 24 [9]. In *Zea mays*, 24 genes that encode PEBP-like proteins are identified, which is divided into three major subfamilies including TFL1-like (six members), mother of ft and TFL1-like (three members), and FT-like based on the protein phylogeny analysis (fifteen members). Protein sequence analysis shows several motifs DPD, PS, HR, and HW are all conserved in the PEBP family [9]. However, due to the limited number of sequences of *pebp* genes in fungi, it is difficult to classify them into groups based on the sequence characteristics and infer their functions.

The overexpression of *Capebp2* in transformant strains during the fruiting stage led to cap redifferentiation, including lamella and intact fruiting bodies. This phenomenon has not been reported before, and more interestingly, both regenerated tissues and intact fruiting bodies were regenerated from the flesh of the fungus. This indicates that the trigger point for regeneration lies in the flesh of the fungus, and further studies are needed to determine whether it is related to the aggregation of the PEBP protein in the flesh. In plants, the transporting path of florigens from the leaf to shoot apical meristem has been well studied. The FT interacting protein 1 (FTIP1) is accompanied by FT proteins moving from companion cells to sieve elements by passive diffusion [46], and other components involved in FT long-distance movement were also uncovered [47,48], which provides a research idea for the study of intracellular production and metastasis of PEBP in fungi.

The phenomenon of cap redifferentiation has been rarely observed in fungi. Environmental signals, oxygen, CO_2_, and light can exert a profound influence on fruiting body development. Moreover, temperature, humidity, volatiles, pH, salinity, and availability of nutrients may play an important role [49,50,51,52]. Light as a signal induces fruiting body formation in many fungi, regulating the primordia formation [53,54] and maturation [55,56]. A red-light receptor FphA represses sexual development [57], while the blue-light LreA/LreB receptor complex stimulates this process [58]. However, the light response genes revealed no correlation with fruiting body structure except stipe bend, and the studies on the cap development are also rarely reported. In *Coprinopsis cinerea*, the *exp1* gene encoding an HMG1/2-like protein is strongly induced in the pileus 3 h before pileus expansion, and the mature fruiting bodies of *exp1* mutant strains normally elongated stipes but with unexpanded pilei remained intact for a long time [59]. In addition, the phenotype of fruiting bodies directly affects the commercial traits of edible mushrooms. To control the phenotype of fruiting bodies through environmental condition changes, it is necessary to clarify the key genes and their molecular regulatory pathways that control the phenotype. Although numerous gene–phenotype combinations have been found in previous studies, there have been no relevant reports on the trait of fruiting body redifferentiation. The regenerative feature requires further clarification of the functional characteristics of the *pebp* genes in basidiomycetes during fruiting body development, including its action and regulation mechanisms.

As important regulation factors, PEBP family proteins are involved in multiple molecular pathways. In *Arabidopsis*, rice, and potato, it was confirmed that the FT and TFL1 proteins bind to the bZIP-type transcription factor FD through the 14–3–3 protein as a co-activator or inhibitor [29,60,61,62]. TFL1-FD inhibits the key regulators CO and GIGANTEA (GI) that promote flowering and repress the expression of FRUITFULL (FUL), LEAFY (LFY), and APETALA1 (AP1), which causes the switch from the branching to flowering state in axillary meristems [28,44,63,64]. These results indicated that the PEBP protein targeting the upstream genes in the molecular pathway regulates the transition from vegetative growth to reproductive stages in plants. In future studies, the environmental factors regulating the PEBP protein and the molecular signaling pathways involved need clarification to explore the function of the PEBP protein family in fungi.

## 5. Conclusions

PEBP family proteins exist widely in biological sources; they regulate various physiological behaviors. However, due to differences in sequence composition, the function of PEBP family proteins has not been revealed in fungi. In this study, a *pebp*-like gene, *Capebp2*, was cloned and identified in the basidiomycetes *C. aegerita*. The sequence analyses showed PEBP were highly conserved in plants and animals, but had low sequence similarity in fungi. However, some conserved motifs, such as DPDAP and HRY, provided references for *pebp* gene discovery and identification in fungi. The transcription investigation showed *Capebp2* may be related to fruiting development reaching a high level in fruiting bodies. The overexpression of *Capebp2* led to cap redifferentiation including intact fruiting bodies and lamella from the flesh of the fungus. These results should provide an important insight for further investigations into the role of the PEBP protein in fruiting body development in *C. aegerita*. However, the production and transportation pathways of PEBP protein, as well as its target and regulatory pathways for fruiting body development, still require further research. In future research, *pepb* gene mining, response to environmental factors, function features, and the regulatory pathways involved need to be elucidated in fungi.

## Figures and Tables

**Table 1:** Primers used in this study.

Primer	Sequence (5′ to 3′)	Description
capebpF	TAACGAATAATAGCCGATATCATGCGTTCTCTCATCCTCTTCATC	Amplification for the whole length of *Capebp2*
capebpR	CCGGTCGGCATCTACGATATCTTAGTCCCCATACCTGAACTTAAAC	
19ha3	TCACCGTAACGAATAATAGCC	Verification of transformants
19ha4	CCCTTATCTGGGAACTACTCAC	
Gpd qF	AGGCTGTCGGCAAGGTTATC	Detection for *gpd* expression level
Gpd qR	TGCGGTGTGACCAATGAAG	
Pebp qF	CAAGCGAAGAAGGCACTAATC	Detection for *Capebp2* expression level
Pebp qR	TAAACGAAGGACTCAGGCG	

## Data Availability

The data presented in the study are deposited in the Supplementary File S1.

## Supplementary Materials

The following are available online at https://www.mdpi.com/article/10.3390/jof9060657/s1, Supplementary File S1 (Sequence characterization: DNA and protein sequence), Supplementary File S2 (Plasmid detection in fruiting bodies).
