# Supplementary material for: Overexpression of the Capebp2 Gene Encoding the PEBP-like Protein Promotes the Cap Redifferentiation in Cyclocybe aegerita
Source: J Fungi (Basel). 2023 Jun 12;9(6):657. doi: 10.3390/jof9060657 (PMC10302294; doi:10.3390/jof9060657)
Supplement: Supplementary file 1 [file jof-09-00657-s001.zip › Supplementary File S1.pdf]

DNA and protein sequence of *Acpebp2* (the introns were marked as gray)

>*C. aegerita*

ATGCGTTCTCTCATCCTCTTCATCGCTACCCTGGTGTCAGCCTTCACCAGCGTTGCGTT
TGCGCAATGGCAAGACACGAACCTCACGGCAGTCAAACTGGCCTTTGAAGAGGCCCA
CGTGCGTTCCCCGTTCCGTCCAGAGTGCATGTCAACCCGCTAAATTATGTGAAATACT
CCAGATAACTCGAGATGTTGGCCTCCCATTCAACCCCCTTGTCATCCTCGGCGTGACC
TACTTCTCCTCCAAAACAGGGCTCATTCCCGTCAAAGCTGGCGCTGAACTCTCCATGA
ACGGTGTGTACAACTTCCTTCCTTCCCCTCTCCTCTTTCTAAAATTTAATTTACAGACA
CCTACTCTGCCCCCACCTTCAACATCGTCGGCAACCCGGGCGTCGGCCCGCTCGTCAT
CTTCTTGATCGACCCGGATGCACCATGGCCGCAAGATCCCTCTCTTGCCCAAGCCCGT
CACTTCATGGGTGGCTCGTACTACTGGGACCCCGAGTCGGGCGTCCTGACAGAAATCT
TCCTGCCTCTCAACCCGTATGATCACCCGCGGCCGTCGAAGGGGTCCGCTCGTCATCG
GTGAGTGCTTCGTTCAGAGCAGAGGATGGTGAAATCCTTGTTGCGTTGCTAACAGATG
TCCATGTTGATAGGTACATCTTCTATGTATTCAAACAATCGGTCGAGCTAGGTCTTCA
AAAGACCATCACCGCGACAAGCTCTCGCCTCAACTTCAACCTGAGCGACTTTGCACG
CGAGACCAACCTCGGAGACCCGATCGGGGGGACCTTCATGTGGATTAGCCCCGACCC
GTAGGACGGACGAGCGGGATGTTAGCTTTTTCGCCTTACTCACAGGTGGTCTCCGAGT
CTTGCACAAGCGAAGAAGGCACTGTACGTTTACTTTGGTGTATGTGCGGCTACTAGAC
TCAGAGTACAGCGCCACAGAATCCATTATTCCTCTAGTGGCTGATAAGGCACCCATG
AACGAATTTGTCCGCGGAATTGCTGCGTACTGGGACCTCTGGAGCTCACGACGCGCC
ACCTGCCCACCATCGCCCGGGGGCGCCACTTCCTCATGGGCAGGCATTACTTTGACTA
CGTTTGGTCCTGTTTCTGGGGTCAGATACCCATCGGCGAGTCTCCGCCTGAGTCCTTC
GTTTAAGTTCAGGTATGGGGACTAA

Protein sequence

>*C. aegerita*

MATDIDAAHVAQLSDSLQRLQVQQDQAITAGQDGEENPGHSSRSPSVPKSPISRDGYGFR
KSGVSTPLMSGQIADGHPHSQLSHELVPDPNGLGWPAKSTISRLNATPEEKAAREKKMATA
VRTILECIGEDPDREGLQRTPERYAQAIMWMTRGYEERLADVINDAIFAEDHDEMVIVRDI
DISSLCEHHLVPFTGKIAIAYIPNQLVLGISKLARIAETFSRRLQVQERLTKQIAIAVQEAIKP
RGVAVVMEATHLCMTMRGVQKPGAITVTSCMLGCFRTQQKTREEFLTLIKH
