# Supplementary material for: Overexpression of the Capebp2 Gene Encoding the PEBP-like Protein Promotes the Cap Redifferentiation in Cyclocybe aegerita
Source: J Fungi (Basel). 2023 Jun 12;9(6):657. doi: 10.3390/jof9060657 (PMC10302294; doi:10.3390/jof9060657)
Supplement: Supplementary file 1 [file jof-09-00657-s001.zip › Supplementary File S2.pdf]

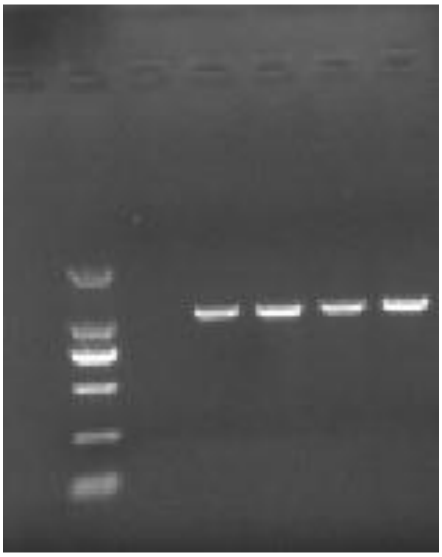

Lane1 2 3 4 5 6

Gel image: plasmid detection in fruiting bodies of four transformants. Lane 1-6: DL2000 marker; wild strain AC0007; T2-1, T2-7, T2-10 and T2-11.
